# How to optimally allocate sampling effort in experimental ecology

**DOI:** 10.1038/s41598-026-38541-4

**Published:** 2026-02-13

**Authors:** Andreas H. Schweiger, Aron Garthen, Michael Bahn, David Chalcraft, Nicolas Schtickzelle, Klaus Steenberg Larsen, Jürgen Kreyling

**Affiliations:** 1 Institute of Landscape and Plant Ecology, Department of Plant Ecology, University of Hohenheim, Ottilie-Zeller-Weg 2, 70599 Stuttgart, Germany; 2 Experimental Plant Ecology, Institute for Botany and Landscape Ecology, Greifswald University, Soldmannstraße 15, D-17487 Greifswald, Germany; 3 Institute of Ecology, University of Innsbruck, Sternwartestr. 15, Innsbruck, 6020 Austria; 4 Department of Biology, East Carolina University, Greenville, NC 27858 USA; 5 Earth and Life Institute, Université catholique de Louvain, Croix du sud 4/L7.07.04, Louvain-la-Neuve, 1348 Belgium; 6 Department of Geosciences and Natural Resource Management, University of Copenhagen, Rolighedsvej 23, Frederiksberg C, 1958 Denmark

**Keywords:** Global change experiments, Ecological models, Experimental design, Non-linear responses, Polynomial fits, Prediction success, Ecology, Ecology, Mathematics and computing

## Abstract

A major aim of experimental ecology is to quantify responses to environmental change. Study designs which optimally capture response patterns are currently debated. A key point in the discussion is how a limited total number of samples should ideally be allocated to replication versus the number of locations along the environmental gradient. Here, we assess how to optimally allocate sampling effort for maximizing prediction accuracy in gradient designs. For this we performed artificial data simulations for different sampling approaches with or without a priori knowledge of the underlying patterns, and applied a set of commonly observed response shapes. Overall, unreplicated sampling with equidistant, systematic placement along the gradient of interest at as many locations or levels as affordable turned out to be the best approach for unknown response shapes. Replication was found to be beneficial when a priori knowledge exists about the underlying, simple (e.g. linear or humped) response shape.

**Supplementary Information:**

The online version contains supplementary material available at 10.1038/s41598-026-38541-4.

## Introduction

A key aspect of ecological research is to quantify ecological responses to environmental change. Ecological responses are often characterized by high degrees of non-linearity, which challenge research approaches^1,2^. Gradient studies, that analyze ecological responses along continuous environmental gradients, have been proposed as the most appropriate approach for detecting response patterns with high prediction accuracy^1,3,4^. Response patterns deduced from such gradient studies may not only advance ecological understanding but can also inform models used for projecting possible future ecosystem responses as an important tool for climate change impact assessments^5^.

**Fig. 1:**
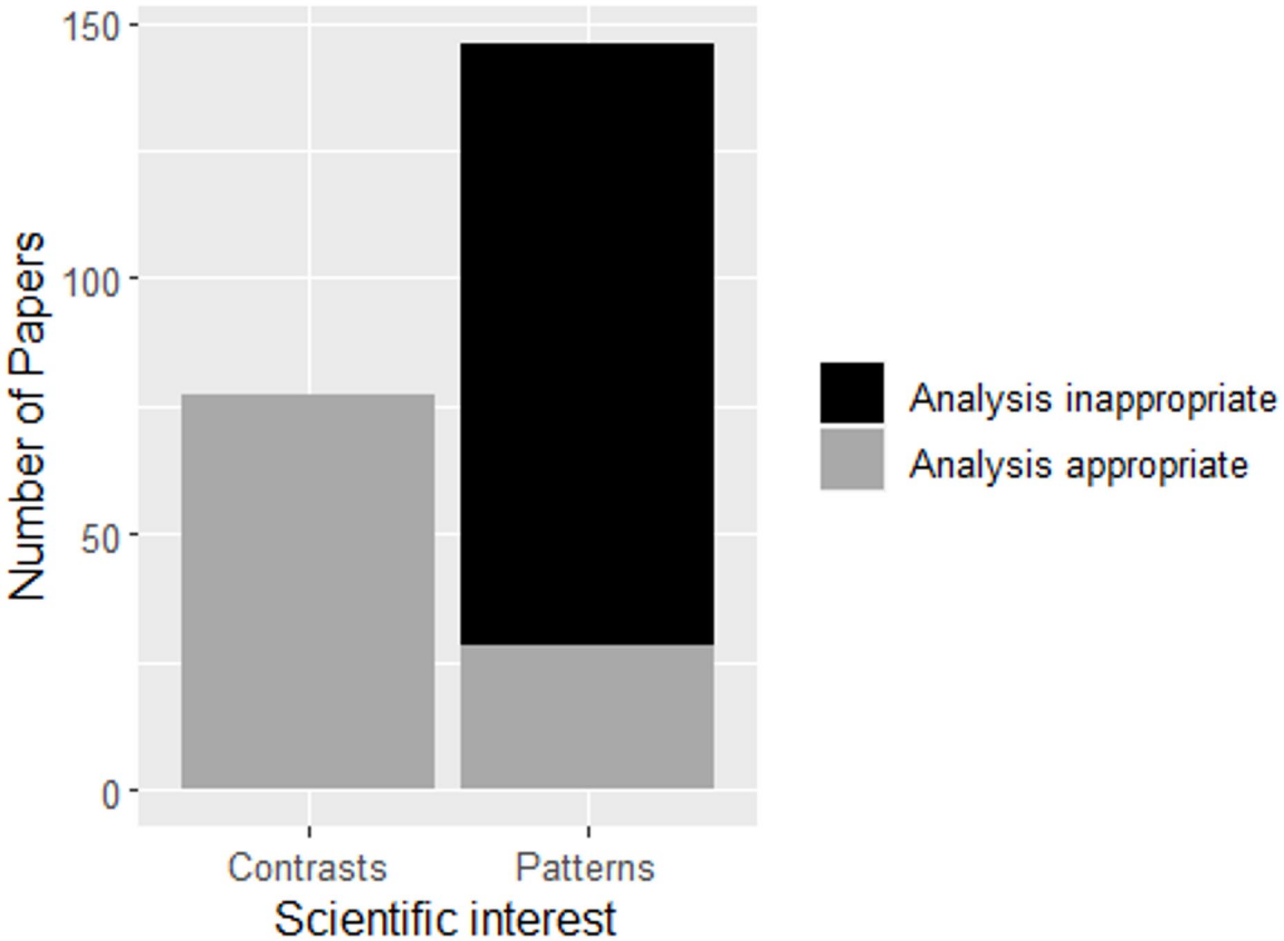
Appropriate and inappropriate analytical approaches chosen in ecological experimentation with interest in either group comparison (contrasts) or response patterns. The analysis was based on an annually stratified random sample of 210 papers published between 1988 and 2022. Grey bars indicate an appropriate use of either contrasts or pattern analysis based on the stated hypotheses and applied statistics, i.e. the black bar indicates papers where pattern analysis would have been the better choice according to the hypotheses, but group contrasts were compared. Detailed methodological description for the systematic literature review is provided in the Supplementary Material (Methods S1).

Gradient designs and analyses are frequently used in subdisciplines of ecology such as vegetation ecology, biogeography or macroecology. However, they remain underrepresented in other fields such as experimental ecology. While the quantification of ecological response patterns is of key interest in experimental ecology, during the last decades researchers have predominately applied suboptimal sampling designs and analytical approaches when studying these responses (i.e. analysis of group contrasts, black bars in Fig. 1 and Figure S1 for temporal development).

Replication is a crucial component of sampling design, especially when contrasts between different groups are analyzed (e.g. in classical treatment vs. control experimental designs). This holds true also for gradient designs, where a high number of replicates for each sampling location along the investigated gradient would be beneficial for prediction accuracy if resources are unlimited for sampling. However, given the limited number of samples which can be realistically taken, an inevitable trade-off emerges between replication and the number of sampling locations which can be considered to cover the environmental gradient of interest. This trade-off has provoked discussions about the optimal sampling procedure, i.e. whether to put more emphasis on the number of sampling locations or experimental levels at the expense of local replication or whether to put more emphasis on replication but with fewer locations or levels sampled along the gradient of interest^1,4,6–8^.

What is known from previous, quantitative studies on the role of replication in gradient studies is that without a priori knowledge of the underlying response shape and random sampling locations along the gradient, unreplicated designs outperform replicated designs in their prediction accuracy for a given sampling effort^1^. Systematic sampling along a gradient, however, can result in an advantage of replicated designs for simple response shapes such as linear or quadratic (humped) relationships when response shape and gradient length are a priori known^6^. Response shape is here defined as the shape of the underlying response along the sampled gradient whereas gradient length is defined as the length of the sampled environmental gradient needed to completely cover the response of interest.

The contrasting perspectives on the importance of replication from these previous studies differ in two aspects, (1) the different metrics of prediction accuracy, (2) differences in sampling strategy, i.e. random placement of sampling locations along the investigated gradient vs. an equidistant, systematic sampling and (3) the assumption on whether the underlying response shape to be analyzed is a priori known or unknown. Based on these apparent differences in previous studies, we assume sampling strategy, i.e. the decision where to locate sampling along the investigated gradient, to be decisive for defining whether replication at the expense of sampling locations has positive or negative effects on prediction accuracy. Sampling strategy has to be furthermore expected to be strongly affected by a priori knowledge of the underlying response shape as well as the length of the investigated gradient. However, understanding the effects of sampling strategy is so far lacking.

**Fig. 2:**
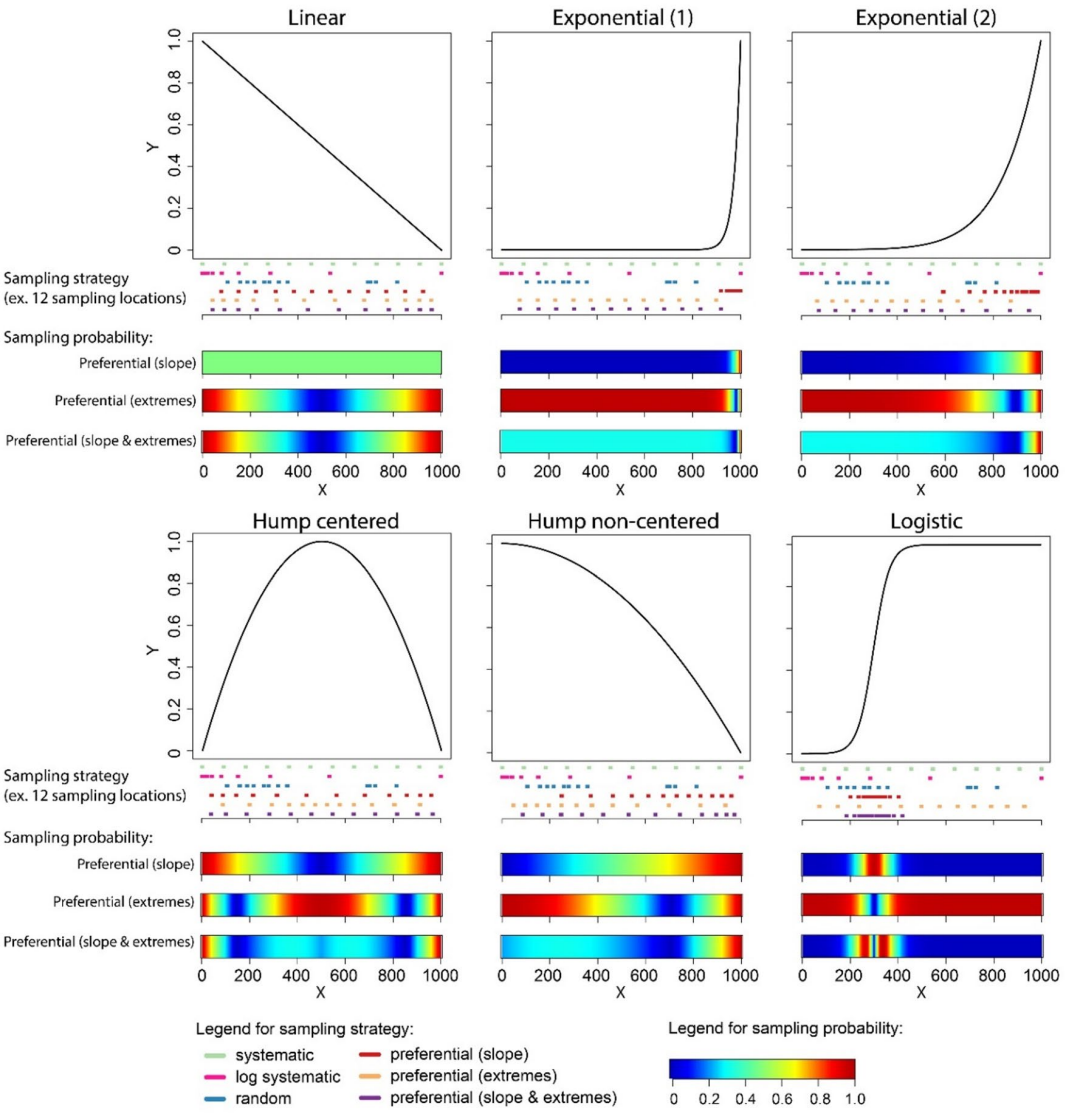
The six response shapes on which the different sampling strategies (i.e. where to place samples along the investigated gradient) and procedures (i.e. how to trade off number of sampling locations against number of replicates per location) were tested in the artificial data simulations. Besides the systematic (systematic and log systematic) and random sampling strategies, preferential sampling strategies that account for critical response points (slopes and/or extremes) were tested, which would in practice only occur with detailed, a priori knowledge of the response patterns. Theoretical sampling probabilities of these three optimized sampling strategies along the studied gradient are shown with the three blue-to-red color bars below each response shape. Additionally, distribution of sampling locations for the six different sampling strategies along the predictor gradient are exemplified below each response shape for 12 sampling locations. Note that the sampling locations for all but the systematic and the log systematic sampling strategies differ slightly (preferential sampling) to strongly (random sampling) between single simulations runs.

To address this lack of knowledge and to reconcile the contrasting perspectives, we extended the artificial data simulations from the previous studies of Kreyling et al.^1^ and Chalcraft^6^ for different sampling approaches, combining different sampling procedures (i.e. how to trade off number of sampling locations against number of replicates per location) and sampling strategies (i.e. where to place sample locations along the investigated gradient) used in regression-type analyses. We tested this for a set of six response shapes representing typical shapes commonly observed in ecology and other disciplines of natural sciences (Fig. 2).

Based on existing evidence from the previous studies, we state four hypotheses which we tested with four corresponding models on our simulation datasets. We hypothesized that (1) higher numbers of replicates to the cost of fewer locations sampled along the gradient decrease prediction accuracy in gradient studies (Model 1 in Table 1). Secondly, we expect the effect of replication on prediction accuracy to interact with the length of the sampled gradient (i.e. predictor extremes included or not, i.e. Model 2 in Table 2). Third, we hypothesize that replication will increase prediction accuracy when the underlying response shape is a priori known and, thus, also the locations of the critical response points along the environmental gradient are known and accounted for in the sampling approach (Model 3 in Table 2). We expected this to be especially relevant for non-linear response shapes, i.e. shapes that cannot mathematically be described as a simple linear model in the form of *y = a∙x + b*. However, we expected replication to yield lower prediction accuracy when the underling response shape is unknown and, thus, critical response points are unknown and therefore not accounted for in the sampling approach. We defined critical response points as locations along the environmental gradient where sampling is crucial for an accurate prediction of the studied response along this gradient. These can be response extremes, i.e. maximum or minimum response values along the studied gradient, or regions of strong response changes, i.e. regions of maximum slope of the response pattern – or a combination of both (Fig. 2). Lastly, we hypothesize that the effect of replication on prediction accuracy increases with total sample size (Model 4 in Table 2).

**Table 1:** Four different models representing the different hypotheses on the effects of replication, total sample size, a priori knowledge on the underlying response shape and length of the predictor gradient on prediction accuracy in gradient studies.

Model	Structure
1	Prediction accuracy ~ # replicates + (1|total sample size) + (1|predictor extremes included) + (1|a priori knowledge)
2	Prediction accuracy ~ # replicates * predictor extremes included + (1|total sample size) + (1|a priori knowledge)
3	Prediction accuracy ~ # replicates * a priori knowledge + (1|total sample size) + (1| predictor extremes included)
4	Prediction accuracy ~ # replicates * total sample size + (1|a priori knowledge) + (1|predictor extremes included)

Terms in parentheses are random effects (random intercepts expressed in the following as 1|x) accounted for in the linear mixed effect models with one or two (interacting) fixed effects.

**Table 2:** Sensitivity of the three measures of prediction accuracy used in this study, i.e. Chalcraft’s prediction success^6^, multiple R^2^ and RMSE.

Noise level and model	Marg *R*²	Marg *R*²	Marg *R*²	Cond *R*²	Cond *R*²	Cond *R*²
	for multiple R2	for Chalcraft	for RMSE	for multiple R2	for Chalcraft	for RMSE
20% noise						
~ replicates	0.03 ± 0.05a	4.8 ∙ 10-3 ± 0.02a	0.01 ± 0.05a	0.42 ± 0.27a	0.07 ± 0.15b	0.12 ± 0.23b
~ replicates*gradient length	0.04 ± 0.05a	4.8 ∙ 10-3 ± 0.02a	0.01 ± 0.05a	0.42 ± 0.27a	0.07 ± 0.28b	0.12 ± 0.23b
~replicates*a priori knowledge	0.26 ± 0.30a	0.02 ± 0.06b	0.04 ± 0.12b	0.38 ± 0.27a	0.07 ± 0.14b	0.12 ± 0.23b
~replicates*total sample size	0.06 ± 0.05a	0.02 ± 0.04a	0.05 ± 0.11a	0.37 ± 0.30a	0.05 ± 0.11b	0.09 ± 0.19b
100% noise						
~ replicates	6.3 ∙ 10-3 ± 0.01a	8.9 ∙ 10-6 ± 1.1 ∙ 10-5a	6.5 ∙ 10-4 ± 2.2 ∙ 10-3a	0.44 ± 0.28a	1.4 ∙ 10-4 ± 1.5 ∙ 10-4b	0.10 ± 0.19b
~ replicates*gradient length	0.01 ± 0.02a	2.3 ∙ 10-5 ± 2.8 ∙ 10-5a	7.1 ∙ 10-4 ± 2.2 ∙ 10-3a	0.45 ± 0.28a	1.5 ∙ 10-4 ± 1.5 ∙ 10-4b	0.10 ± 0.19b
~replicates*a priori knowledge	0.32 ± 0.32a	3.1 ∙ 10-5 ± 2.5 ∙ 10-5b	0.01 ± 0.03b	0.37 ± 0.29a	1.4 ∙ 10-4 ± 1.5 ∙ 10-4b	0.10 ± 0.18b
~replicates*total sample size	0.04 ± 0.05a	4.4 ∙ 10-5 ± 5.9 ∙ 10-5b	0.05 ± 0.12b	0.43 ± 0.29a	7.2 ∙ 10-5 ± 6.7 ∙ 10-5b	0.07 ± 0.15b

Depicted is the sensitivity of prediction accuracy, i.e. Amount of variation explained by different combinations of simulation settings (i.e. Sampling procedure and strategy) realized by different mixed effect models for different levels of random noise in the response (i.e. 20% and 100%). Differences in the explanatory power of the different sets of predictors (i.e. Number of replicates, gradient length, a priori knowledge on the underlying response shape and total sample size) were tested based on marginal R^2^ (Marg R^2^) values for the different measures of prediction accuracy with significant differences being indicated by different letters. Significant differences in the sensitivity of the different measures of prediction accuracy were tested based on conditional R^2^ (Cond R^2^) and are indicated by different letters. The effects of gradient length are quantified as predictor extremes being ex-/or included while the effects of a priori knowledge have been quantified and response pattern is a priori known or unknown. For more details see the methods section as well as tables S1 to S6.

Based on the results of our analyses, we derive recommendations on how to optimize sampling approaches in observational and experimental ecological research, including when to replicate and when to apply non-replicated study designs. Furthermore, we provide recommendations on optimized sampling strategies, i.e. where to best sample along gradients.

## Methods

### Artificial data simulations

We performed simulations based on artificial data similar to Schweiger et al.^4^. To be fully comparable to Chalcraft^6^, we focused on one-factorial responses. We varied four parameters in our simulations: (1) response shape (i.e. mathematical function underlying the observable response along a gradient of environmental conditions), (2) sampling procedure (i.e. total sample size and how to trade off number of sampling locations against number of replicates per location along the environmental gradient), (3) sampling strategy (i.e. where to place sampling locations along the investigated gradient), and (4) level of stochasticity in the response.

We simulated six different linear to highly nonlinear response shapes representing typical and varied shapes frequently used in ecology and other disciplines of natural sciences to describe response patterns (Fig. 2). Each response shape simulates a response variable (y; e.g. any numeric biotic variable such as photosynthetic activity, species richness or population viability) in response to a numeric environmental driver (x; e.g. temperature, water availability, soil pH or nutrient availability). The specific responses of y to changes in x were formulated as linear or nonlinear functions of the form y_i_ = f_i_(x_i_)^1,4,6^. For transferability and to allow general conclusions, we scaled our variables in arbitrary units^1^; cf. Schweiger et al.^4^.

Different sampling procedures were realized by systematically varying the number of total samples drawn from the underlying response shape and the number of locations at which these samples were placed. We used 6 to 96 samples to cover the range of total sample sizes commonly realized in univariate ecological experiments. This total number of possible observations can either be used for covering the study gradient with many sampling locations and, thus, reducing (or completely abolishing) replicates at each sampling location, or on the contrary for increasing the number of replicates sampled from fewer sampling locations along the gradient. A single sample at a location corresponds to no replication, irrespective of the total number of samples drawn, because we define replicates as the number of samples taken at a single sampling location along the driver gradient^1^; cf. Schweiger et al.^4^. Our sampling procedures are therefore combinations of 3 to 96 locations sampled and 1 to 32 replicates per location.

We considered six sampling strategies, differing in the way the number of sampling locations were placed along the driver gradient. Besides random selection of sampling locations (cf. Kreyling et al.^1^ and a systematic, equidistant sampling (with the two ends of the gradient always sampled; cf. Chalcraft^6^, we applied a set of four preferential sampling strategies to account for critical response points. Examples for sample placement for each sampling strategy are given in Fig. 2. Criticality of sampling locations was quantified (1) as the slope of the respective response curves at the specific location (the higher the slope at a particular location the higher the criticality of the response value at this location), (2) as the extremeness of the response value at a specific location (relative to the arithmetic mean of the minimum and maximum of all response values along the studied gradient), and (3) as a combination of both factors equally weighted. These criticality values were standardized and used to quantify sampling probability of the locations along the investigated gradient in an adjusted, randomized sampling. To account for a non-linear scaling of the environmental gradient, we additionally applied a log systematic sampling strategy, where we sampled equidistantly distributed on a log-scale along the driver gradient. Such non-linear scaling of the driver gradient is a common feature e.g. in water or light response curves conceptualized in ecophysiological reaction norms. Preferential sampling as realized in these simulations might appear to be unrealistic in practice when a priori knowledge on the underlying response shape is entirely missing. However, such a priori knowledge can be obtained from pre-studies or literature, allowing for the design of preferential sampling strategies also under real-world conditions.

Different levels of stochasticity in the response variable were tested for all different sampling procedures and strategies applied to the different response shapes by allowing the sampled response values (y) at each sampled location to scatter around the ‘true’ response values with a normal distribution corresponding to 20% (i.e. c. 85% percentile) or 100% (100% percentile) of the absolute response value at this location (cf. Kreyling et al.^1^. Information about the levels of random noise in ‘real-world’ data is extremely rare. Richardson et al.^9^ estimated random noise to reach a maximum of 23% of total variation for eddy flux measurements – a highly uncertain method in environmental science. Kelly et al.^10^ reported similar levels of random noise for assessments based on species community composition, which ranged between 3 and 22% of total variation (on average 11.3 ± 4,6%). Based on these observations, we assume our 20% noise scenario as a close-to-real world scenario, which will be of practical use in many situations, whereas our 100% noise scenario has to be considered as a very extreme, ‘high-noise’ scenario.

### Analysis of simulations

We used polynomial regression for pattern prediction and interpolation to be fully comparable to Chalcraft^6^. For the unknown response shape scenario, we allowed the algorithm to choose the best fitting model for the sampled test data from a set of polynomial equations (1st to 4th order) based on minimal AIC. For the scenario where we assumed the response shape to be a priori known, we selected the prediction model which represented the respective response shape. For each test data set/each selected prediction model, we checked the ability to reveal the true underlying response shape by quantifying prediction accuracy through plotting predicted against the true response values. We quantified prediction accuracy by using the two methods of Kreyling et al. and and Chalcraft^1,6^ plus an additional measure for prediction accuracy based on root mean square error (RMSE) to achieve a balanced view on different perspectives on prediction accuracy and compared the three approaches. According to Kreyling et al.^1^, we quantified prediction accuracy as the deviation between the response shape obtained from the predicted response based on the different sampling strategies and procedures and the ‘true’, known underlying response shape, i.e. multiple R^2^ of a linear regression between predicted and ‘true’ response values based on a fixed number of 1000 equidistantly spaced locations. Multiple R² thereby measures how closely predicted values fall along a line, which describes how predicted and true values covary with each other.

Chalcraft^6^ criticized multiple R² for not measuring the degree to which predicted and true values match, but only the degree to which they correlate. We therefore pursued an alternative approach and forced the regression between predicted and ‘true’ response values through zero and on a slope of one, measuring the degree to which predicted and true values perfectly match (see Chalcraft^6^ and simulation R code in the supplementary material). In the third approach, we quantified the root mean square error (RMSE) based on the predicted and ‘true’ response values for a fixed number of 1000 equidistantly spaced locations. For better comparison with the multiple R^2^ and Chalcraft approaches, where higher values indicate higher prediction accuracy, we here used negative RMSE as the measure of prediction accuracy. RMSE shows a linear relation to increasing noise, which is in contrast to the non-linear behavior of multiple R^2^ and Chalcraft’s prediction success (Supplementary Material Figure S2). To evaluate the success of revealing the known underlying response shapes, we repeated sampling 1000 times for each combination of total number of experimental units, number of locations and number of replicates. To test our different hypotheses, we analyzed the output of our simulations by comparing four different models (see Table 1). For each hypothesis and the corresponding model, we accounted for the effects of all four predictors (i.e. number of replicates, gradient length, a priori knowledge of underlying responses shape and total sample size) to maintain total explanatory power of the models. We therefore added the other predictors not in the focus of the respective hypothesis as random terms to the model.

Gradient length covered by sampling locations can potentially influence the quantification of the prediction accuracy, as longer gradients tend to result e.g. in higher R². Furthermore, real ecological gradient studies might lack knowledge about total length of the driver gradient. To account for the effect of the sampled gradient length on the accuracy to predict the response of interest, we repeated all simulations with the two ends of the driver gradient always being sampled (scenario: “with predictor extremes”). Effects of replication on prediction accuracy were tested using robust linear mixed effect models with total sample size and response shape as random effects (random intercept). Gradient length (i.e. with and without predictor extremes) as well as the existence (or lack) of a priori knowledge of the underlying response shape were tested either as fixed effects in interaction with replication or as random effects (random intercepts expressed in the following as 1|x) as formulized in the four different models (Table 1), implemented using the lmer()- command of the *lmerTest*-R-package (v.3.1-3.1; Kuzentsova et al.^11^. For each model, we calculated marginal and conditional R² using the r.squaredGLMM() function of the *MuMIn*-R-package (v. 1.43.17; Barton^12^. Difference between the models in marginal and conditional R² were tested by Analysis of Variance with a post hoc multiple comparison test. For each model, the relative contribution of each individual predictor as well as the shared contribution of interacting predictors were quantified using the variation partitioning approach proposed by Legendre^13^ based on marginal R^2^. All simulations and analyses were performed in R^14^ with a level of significance being set to alpha = 0.05.

## Results

Comparing the three measures of prediction accuracy, multiple R^2^ showed significantly higher sensitivity to variations in the different simulation settings than RMSE or Chalcraft’s prediction success. Conditional R^2^ values of the four different models testing the effects of replicate numbers, gradient length, total sample size and a priori knowledge on the underlying response shape were significantly higher for multiple R^2^ than for RMSE or Chalcraft’s prediction success (see Table 2 as well as Supplementary Material Figure S3 and S4 as well as Tables S1 to S6 for more details). In the following we will show and discuss the results obtained for all three measures of prediction accuracy.

Prediction accuracy differed among the different sampling strategies with systematic sampling yielding highest prediction accuracy. This was consistent across the three different measures of prediction accuracy, the different noise levels as well as independent of a priori knowledge of the underlying response pattern (Supplementary Fig. S5-S7 as well as Table S8-S10).

The number of replicates in combination with the a priori knowledge on the underlying response shape (i.e. Model 3) turned out to be the best set of explanatories for prediction accuracy based on multiple R^2^ (see Marginal R^2^ for 20% and 100% noise in Table 2). The other three sets of predictors describing prediction accuracy, i.e. *replication* (i.e. Model 1), replication * gradient length (i.e. Model 2) and replication* total sample size (i.e. Model 4) explained significantly less variation in prediction accuracy (i.e. multiple R^2^) and were not statistically different from each other (*p* > 0.05 in multiple comparison test of Marginal R^2^ values, see Table 2). For RMSE and Chalcraft’s prediction success, all models showed very low explanatory power with no significant differences between the different models, except for Chalcraft’s prediction success at 100% noise, where the replication-only model (i.e. model 1) explained significantly less variation than the other models where replication interacted with the other explanatories. Details on the individual model statistics for the different sampling strategies and response shapes are available in Table S1 to S6.

For the best explanatory model based on multiple R^2^ (i.e. Model 3), replication turned out to be the main explanatory of prediction accuracy, with 23 ± 31% (arithmetic mean ± standard deviation) of explained total variation for 20% of noise and 31 ± 32% for 100% of noise (Figure S8 and S11). Explanatory power of a priori knowledge of the underlying response shape and its shared predictive power with replication was significantly lower (i.e. 2.0 ± 4.2% with 20% noise as well as 0.70 ± 1.3% for 100% noise; in all cases *p* < 0.001 in multi-comparison tests). For RMSE and Chalcraft’s prediction success, relative contributions of replicates, a priori knowledge of the underlying response shape and their combination were all minor (see Supplementary Figures S9, S10, S12 and S13).

**Fig. 3:**
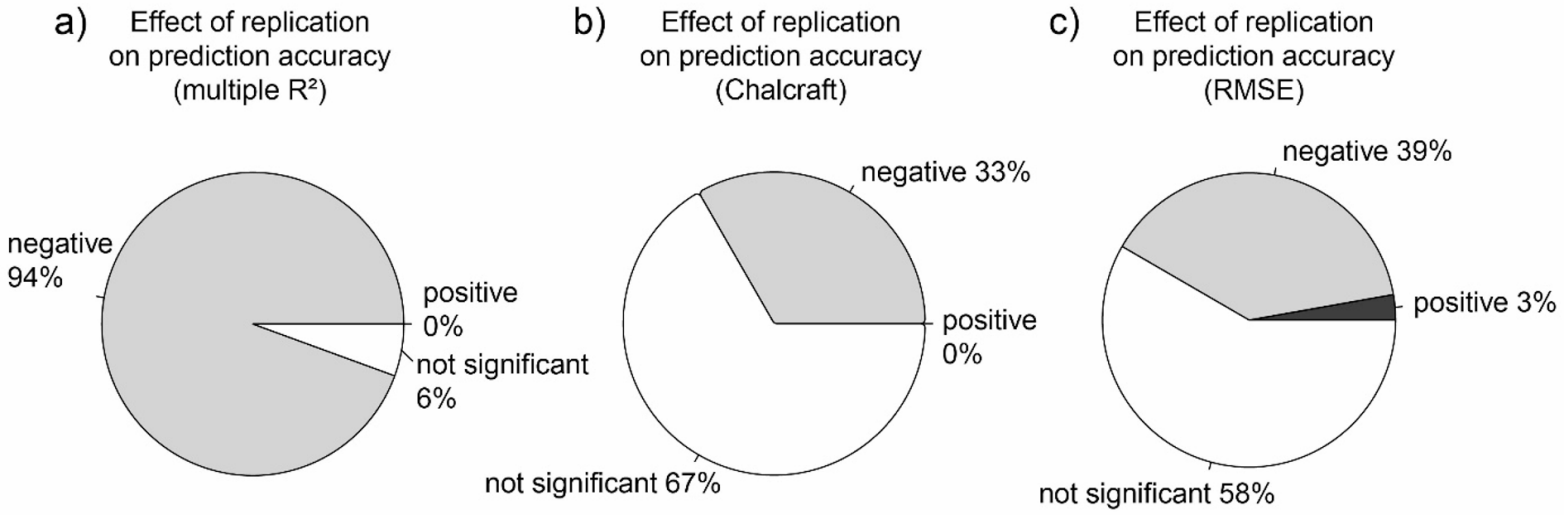
Effect of replication on prediction accuracy for a given number of samples. Effects were quantified for prediction accuracy measures based on multiple R^2^ (**a**), Chalcraft’s prediction success, (**b**) and negative RMSE (**c**) for 20% noise. A priori knowledge of the underlying response shape was accounted for in a linear mixed effect model (Model 3 in Table 1). Results for 100% noise are summarized in Fig. S11.

For multiple R², replication showed significant negative effects on prediction accuracy for 94% of all tested cases (i.e. combinations of the different response shapes, sampling procedures and sampling strategies) at 20% noise. These percentages were visible lower for Chalcraft’s prediction success, i.e. 33% and RMSE, i.e. 39% of all cases tested (see Fig. 3 and Table S7 for details). Positive effects of replication were detected in 0% (R^2^ and Chalcraft) and 3% (RMSE), whereas non-significant effects were observable in 6, 58 and 67% of all tested cases for multiple R^2^, RMSE and Chalcraft’s prediction success, respectively. Similar patterns were observable for 100% of noise despite for Chalcraft’s prediction success, for which no significant effects of replication were observable (see Supplementary Fig. S14 and table S7 for more details).

## Discussion

### Summary of findings

High prediction success in gradient analyses is determined by two factors: (1) identification of the response shape and (2) precise estimation of model parameters to maximize predictive accuracy across a wide range of environmental conditions. To achieve this, sampling has to be optimized under limited resources, thus, limited total sample size. For gradient studies, we have shown in this study that available resources should be invested into increasing the number of sampling locations at the expense of replication when the underlying response shape is unknown or complex. Nevertheless, replication can be beneficial in gradient studies when the response shapes are simple and known.

Our simulations have furthermore shown that systematic sampling along the gradient of interest generally outperforms all other tested sampling strategies, except for complex, a priori known response shapes, for which preferential sampling of critical response points (i.e. local extremes or parts of the gradient with strong changes) can be beneficial. This finding might be a major relief for researchers who are worried how to best sample responses along environmental gradients or decide upon experimental treatment levels without a priori knowledge on the underlying response shapes.

### Relation to previous work

Previous quantitative studies on the role of replication have shown that replication is not necessary or can even have negative effects on prediction accuracy in scenarios where the underlying response patterns are unknown^1^. Systematic sampling along a gradient, however, can result in an advantage of replicated designs for simple response shapes such as linear or quadratic (humped) relationships when response shape and gradient length are a priori known^6^.

By including a priori knowledge of the response shape in our simulations, we were able to reproduce the findings of Chalcraft for the response shapes he investigated (i.e. linear and centered hump). This resolves the apparent discrepancy between the two studies of Kreyling et al.^1^ and Chalcraft^6^ by showing that existing or lacking a priori knowledge on the underlying response shape is decisive for whether replication is beneficial for increasing prediction accuracy or not.

**Fig. 4:**
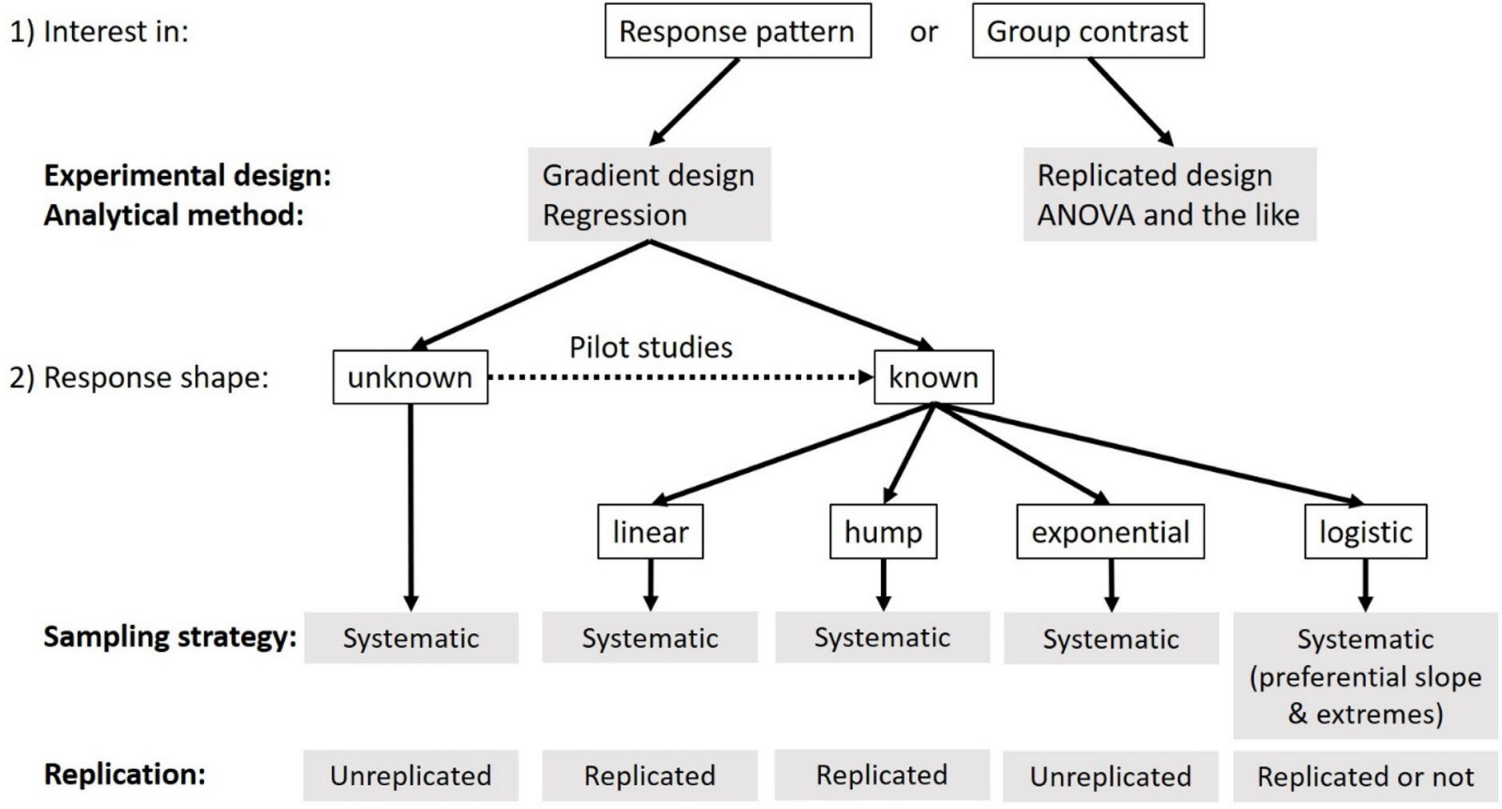
Decision tree for ecological experimenters based on general considerations and conclusions drawn from the integration of simulation results for all three measures of prediction accuracy (Tab S4 to S7). Individual decision trees for each of the three measures of prediction accuracy are provided in the Supplement Figures S15 to S17.

Kreyling et al.^1^ already pointed out that replicated designs in combination with classical ANOVA analyses are preferable when researchers are interested in differences among treatment groups. Replications were furthermore highlighted to be beneficial when studies focus on predicting gradient responses with high local precision or when predicting local variation of gradient responses (see Table 2 in Kreyling et al.^1^. Our present study showed that unreplicated designs yielded higher prediction accuracies when the response shape was unknown or the known response shape was more complex (i.e. exponential or logistic). Replication turned out to be beneficial when the underlying response shape was a priori known and rather simple (i.e. linear or hump, see Fig. 4 for a summary). We detected a positive interaction between replication and knowledge on the underlying response shape, meaning that the negative effect of replication tends to be higher for unknown response shapes and the negative effect of missing a priori knowledge about the underlying response shape is stronger for stronger replication. However, this interactive effect was rather weak (see Fig S8a, S9a and S10a).

Replication obviously increases the prediction accuracy for any specific location along the environmental gradient in the presence of (white) noise by providing a better estimate through averaging. This is especially crucial when aiming for contrasts between factorial groups such as two-level manipulation experiments and when data variance for specific treatment conditions (i.e. within different treatment groups) should be minimal to increase predictive accuracy at the single location^6,15^. Such classical, replicated experiments are inevitable whenever binary environmental drivers are tested such as presence or absence of specific species or functional groups or the effects of sites or management schemes differing non-continuously or along unknown gradients^1^. However, classical experimental and analytical approaches, such as a two-level manipulation of environmental factors, have been already highlighted in previous studies to be inappropriate when aiming for characterizing such non-linear processes regulating ecosystem responses to multifactor drivers of global change^16^ or to quantify phenotypic plasticity^17^.

Besides their advantages for mechanistic understanding and model extrapolation, gradient designs are capable of capturing non-linear responses that often characterize ecological responses to global climate change^18–20^. Unreplicated gradient designs can furthermore help to avoid pseudo-replication in less controlled settings such as field experiments or other empirical investigations along environment gradients^21^, a common criticism of replicated designs under such conditions^22^.

### Implications

Our simulations showed that systematic sampling generally outperforms other sampling strategies including preferential sampling. Still, it might be difficult to implement systematic, equidistant sampling in practice, as scaling between the investigated driver and response might often be non-linear (e.g. metabolic rates double every 10 degrees of temperature increase, or biological/ecological responses to increasing precipitation will often be log-scaled). Under such circumstances it might be not entirely clear what equidistant exactly means and, thus, whether linear or non-linear response shapes must be assumed or predictor values have to be transformed to obtain linear scaling. In such cases, random placement of samples along the investigated gradient might provide an alternative solution when the location of critical response points is unknown and therefore cannot be accounted for in a preferential sampling strategy. Surprisingly, even if the underlying pattern is known, systematic sampling performed best, or at least not systematically worse, than preferential sampling designs. Preferential sampling covering critical response points such as local extremes or parts of the gradient with strong changes (steep slopes) can become especially beneficial for prediction accuracy when the response shape is a priori known (i.e. logistic response shapes in Fig. 4). A priori knowledge on the investigated response shape – which can be obtained from pilot studies or might be inferred from existing literature – can furthermore significantly increase sampling efficiency and prediction accuracy in subsequent studies (see Supplementary Table S8-S10). Based on the advanced understanding emerging from our simulations, we derived a set of recommendations to optimize sampling in ecological research (Fig. 4).

### Limitations & future directions

By tackling a wide range of different settings in our simulations, we consider our analyses representative for settings of sampling strategies and procedures commonly used in ecological research focusing on gradient analyses. In our analysis, replication turned out to be very strong explanatory of prediction accuracy. However, variation in explanatory power was high among the different response shapes and sampling strategies. Furthermore, the strong, individual effect of replication on prediction accuracy vanished when combined with gradient length (Model 2) or total sample size (Model 4) as interacting predictors (see Figures S8 and S11). Depending on the length of the gradient along which responses are measured and analyzed, different response shapes might be identified as most accurate to describe the underlying response. This phenomenon of contrasting response patterns being identified for the same underlying process is reported for well-known, functional relationships such as the biodiversity-productivity relationship and can cause misguided discussions about the underlying mechanisms^23^. In this simulation we focused on polynomial equations (1st to 4th order) for model fitting to be completely comparable to the previous studies by Kreyling et al. and Chalcraft^1,6^. Other modelling approaches such as splines or GAMs could be tested in future studies although misidentification of response shapes which originate from incomplete sampling might get even more likely with more flexible models.

Although our simulations covered variation in gradient length (i.e. scenarios with and without predictor extremes), we did not explicitly tackle the topic of gradient sampling beyond commonly considered ranges – a fact that calls for future studies in this direction. One empirical approach to resolve this phenomenon of incomplete gradient sampling might be to enlarge the range of investigated, environmental conditions into extreme conditions even beyond the biological limits of the studied responses^5^; e.g. species-specific mortality; Kreyling et al.^24^. Usually, experiments keep the range of investigated conditions within conservative boundaries, presumably because more extreme (although potentially realistic) treatments may have a catastrophic impact on a studied organism or ecosystem, which potentially results in the loss of costly replicated samples due to e.g. death of organisms when physiological limits are crossed under extreme environmental conditions^16^. Unreplicated gradient designs will allow for such extensions into the extremes without losing too many samples when model misspecification emerging from highly nonlinear responses towards extreme values can be ruled out. Gradient studies that realize a wide range of environmental conditions will furthermore provide understanding on how far a certain response of a specific organism or ecosystem is situated relative to its lower or upper tolerance limit^16^. Unreplicated designs will serve for covering investigated gradients more densely and for pushing experimental systems beyond historical and forecasted extremes. The latter will be decisive for global change impact research, as it enhances our understanding of stressor–response relationships and thresholds in state and impact beyond already realized environmental conditions.

## Supplementary Information

Below is the link to the electronic supplementary material.

Supplementary Material 1

Supplementary Material 2

Supplementary Material 3

Supplementary Material 4

## Data Availability

All data and scripts for data simulation and analyses are freely available on Zenodo https://doi.org/10.5281/zenodo.10134667.
