# Supplementary material for: How to optimally allocate sampling effort in experimental ecology
Source: Sci Rep. 2026 Feb 13;16:6503. doi: 10.1038/s41598-026-38541-4 (PMC12910100; doi:10.1038/s41598-026-38541-4)
Supplement: Supplementary file 4 — Supplementary Material 4 [file 41598_2026_38541_MOESM4_ESM.docx]

**Supplementary Material for Schweiger et al. “How to optimally allocate sampling effort in experimental ecology”**

**Methods S1**

***Quantitative literature survey***

We conducted a quantitative literature survey to evaluate the general importance and implementation of gradient studies in ecological research, i.e. the question whether ecologists are interested in group contrasts or in response patterns. We define group contrasts as comparisons of two or more factorial groups or distinct treatment levels such as e.g. different amounts of fertilizer added, which includes classical treatment vs. control designs. We define response patterns as patterns of a target response variable along a continuous gradient such as natural or experimental environmental gradients. This question was answered based on the specific research question(s) and/or hypothesis(es) stated in the respective paper. Based on this, we furthermore asked whether the researchers chose the appropriate analytical approach to answer their question, i.e. using group comparisons such as Student’s t-test or ANOVA when interested in group contrasts or using regression approaches when interested in response patterns. We screened ecological literature from 1988 to 2022 by searching Web of Science on Jan 31, 2022 with the topic “experiment* not model” refined by document type “article” and the web of science category “ecology”. Of the 37690 papers returned by this search, we randomly selected six papers per year, adding up to a total of 210 papers over 35 years. Studies were classified as “Analysis inappropriate” if the analytical approach (group comparison vs. regression) did not match the study question (interest in group contrasts vs. response patterns) and as “Analysis appropriate” if it matched. In case of papers with various research questions and experiments belonging to different cases, these papers were assigned to both groups.


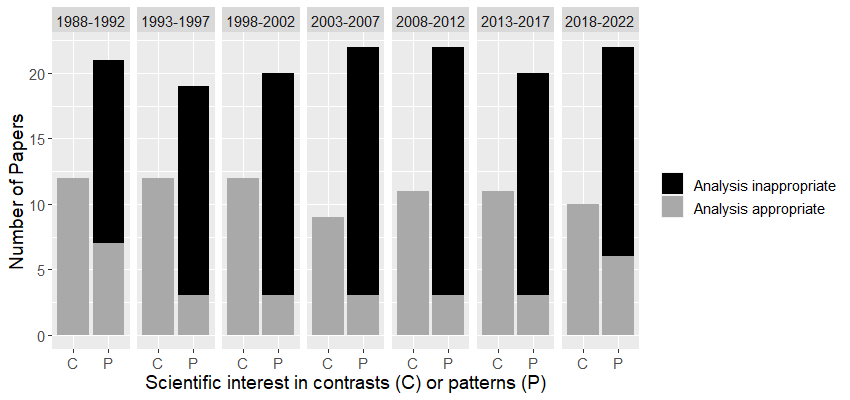
***Figure S1.*** *Temporal development of the portion of appropriate and inappropriate analytical approaches chosen in ecological experimentation with interest in either group comparison (contrasts) or response patterns. The analysis was based on an annually stratified random sample of 210 papers published between 1988 and 2022. Grey bars indicate an appropriate use of either contrasts or pattern analysis based on the stated hypotheses and applied statistics, i.e. the black bar indicates papers where pattern analysis would have been the better choice according to the hypotheses, but group contrasts were compared. This discrepancy between study interest and analytical approach (i.e. interests in response patterns analyzed with inappropriate analytical tools such as analysis of group contrasts) did not change over time (R²=0, p=0.856).*


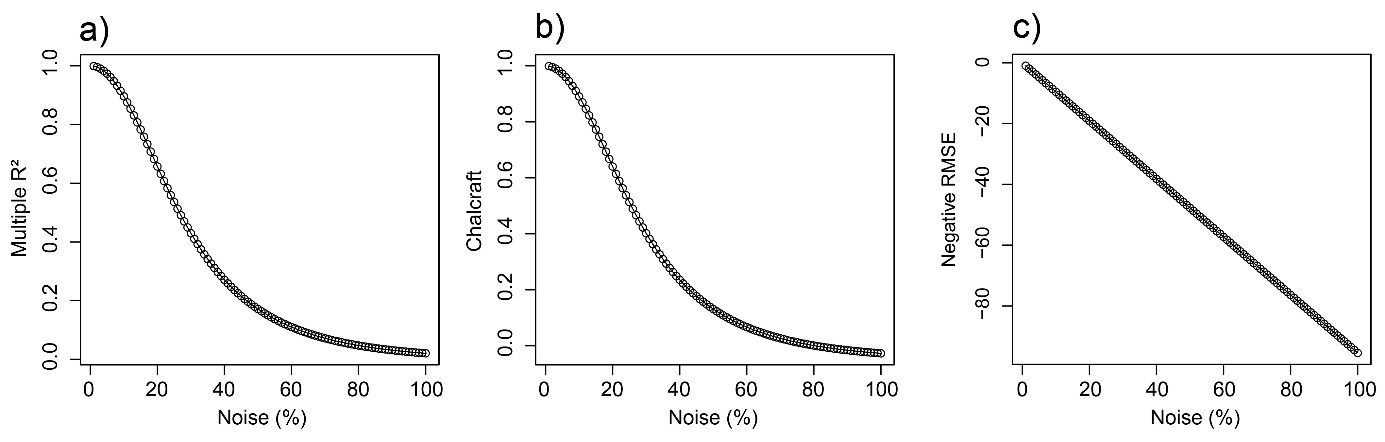


***Figure S2.*** *Response of Multiple R^2^ (a), Chalcraft’s measure for prediction success (b) and RMSE (negative values, c) to increasing random noise and with a linear response shape underlying.*


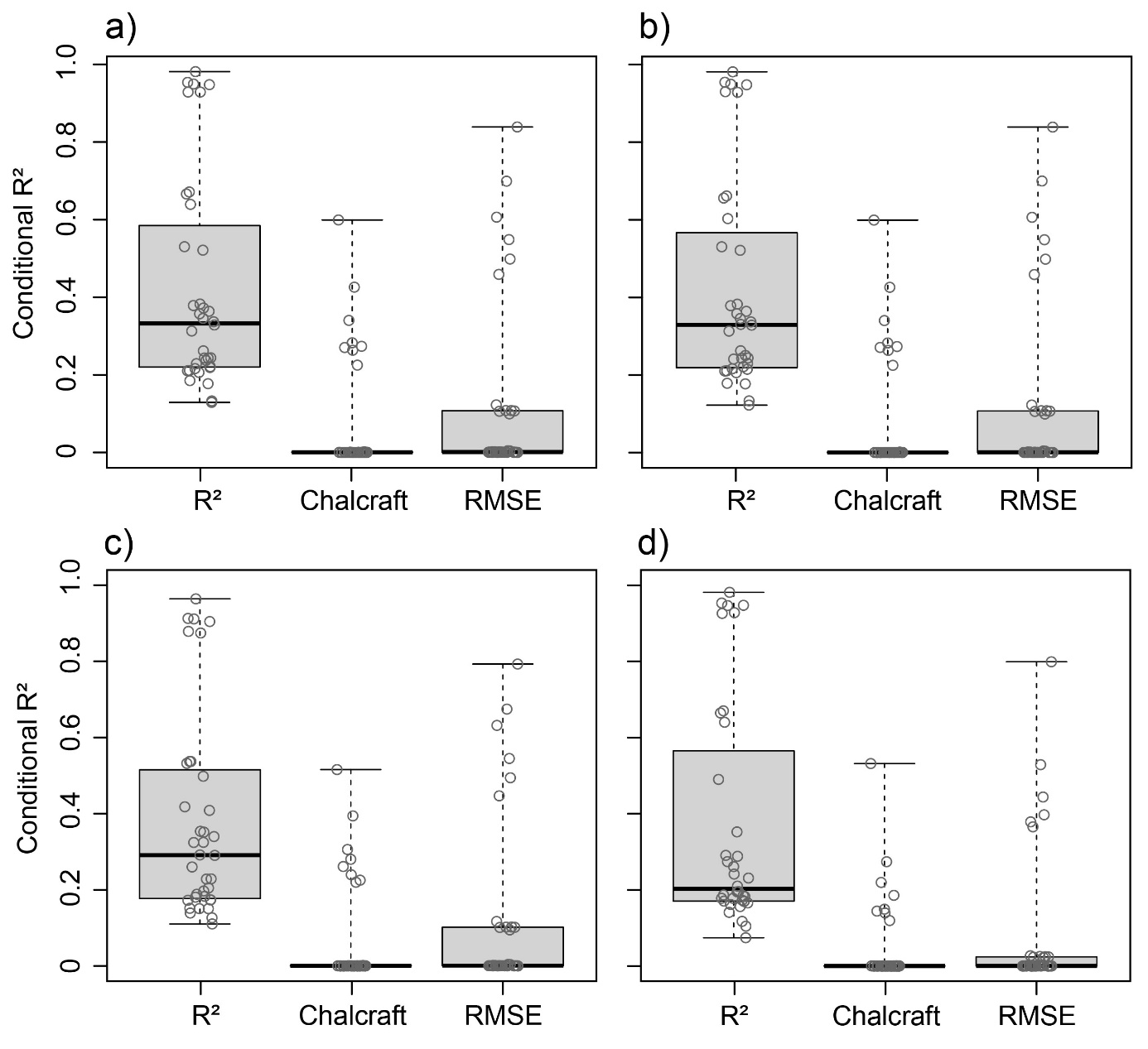


**Figure S3.** *Sensitivity of the three measures of prediction accuracy applied in this study (i.e. multiple R^2^, Chalcraft’s prediction accuracy and negative RMSE) for four different models reflecting the different simulation settings (i.e. sampling effort, sampling strategy and sampling procedure), a) model 1: Prediction accuracy ~ number of replicates + (1|total sample size) + (1|predictor extremes included [yes/no]) + (1|a priori knowledge on the underlying response shape [yes/no]), b) model 2: Prediction accuracy ~ nbr of replicates * predictor extremes included + (1|total sample size) + (1| a priori knowledge on the underlying response shape), c) model 3: Prediction accuracy ~ nbr of replicates * a priori knowledge on the underlying response shape + (1|total sample size) + (1|predictor extremes included), d) model 4: Prediction accuracy ~ nbr of replicates * total sample size + (1|predictor extremes included) + (1|a priori knowledge on the underlying response shape). Based on 20 % of random variation (noise) in the response.*


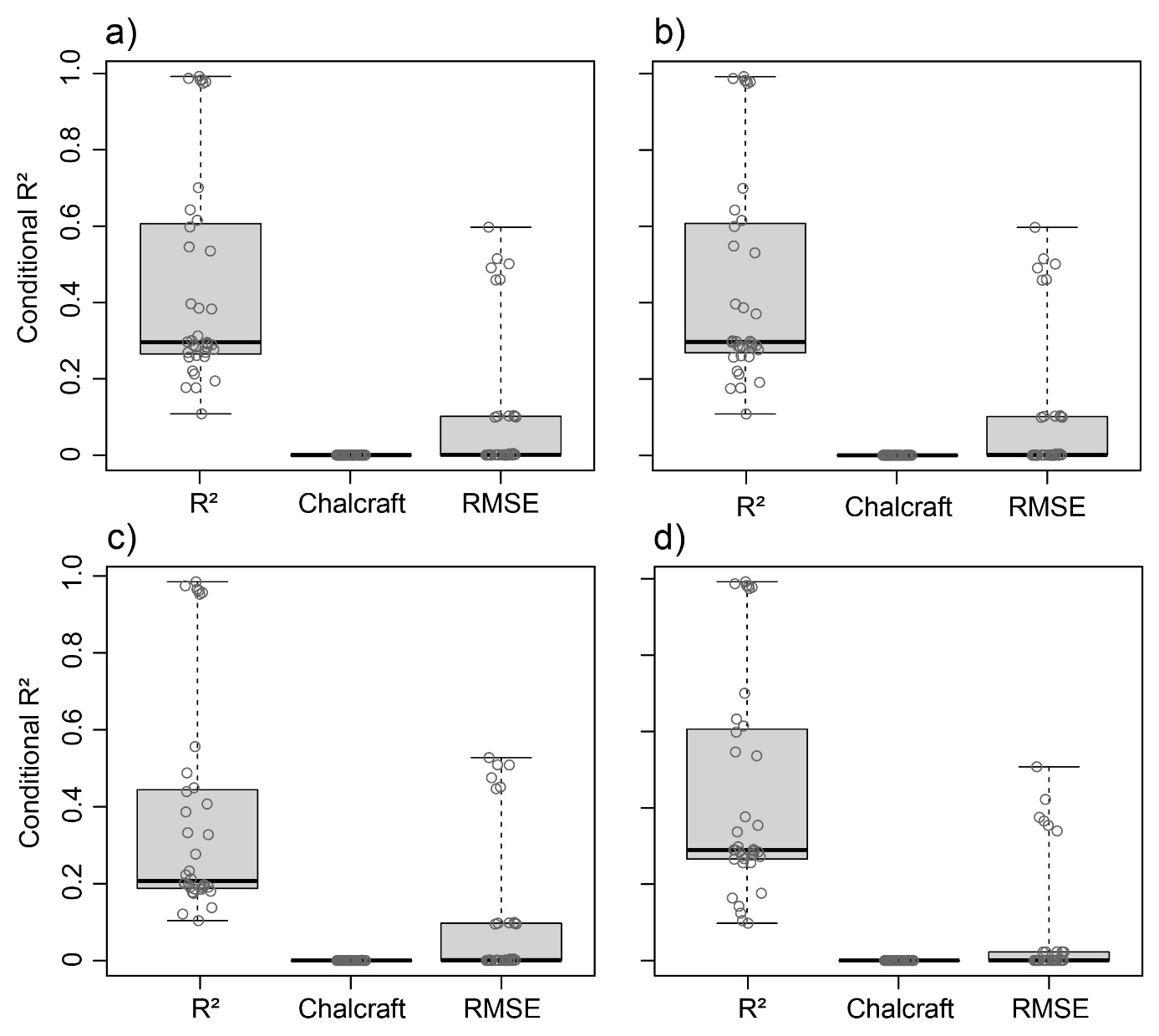


**Figure S4.** *Sensitivity of the three measures of prediction accuracy applied in this study (i.e. multiple R^2^, Chalcraft’s prediction accuracy and negative RMSE) for four different models reflecting the different simulation settings (i.e. sampling effort, sampling strategy and sampling procedure), a) model 1: Prediction accuracy ~ number of replicates + (1|total sample size) + (1|predictor extremes included [yes/no]) + (1|a priori knowledge on the underlying response shape [yes/no]), b) model 2: Prediction accuracy ~ nbr of replicates * predictor extremes included + (1|total sample size) + (1| a priori knowledge on the underlying response shape), c) model 3: Prediction accuracy ~ nbr of replicates * a priori knowledge on the underlying response shape + (1|total sample size) + (1|predictor extremes included), d) model 4: Prediction accuracy ~ nbr of replicates * total sample size + (1|predictor extremes included) + (1|a priori knowledge on the underlying response shape). Based on 100 % of random variation (noise) in the response.*


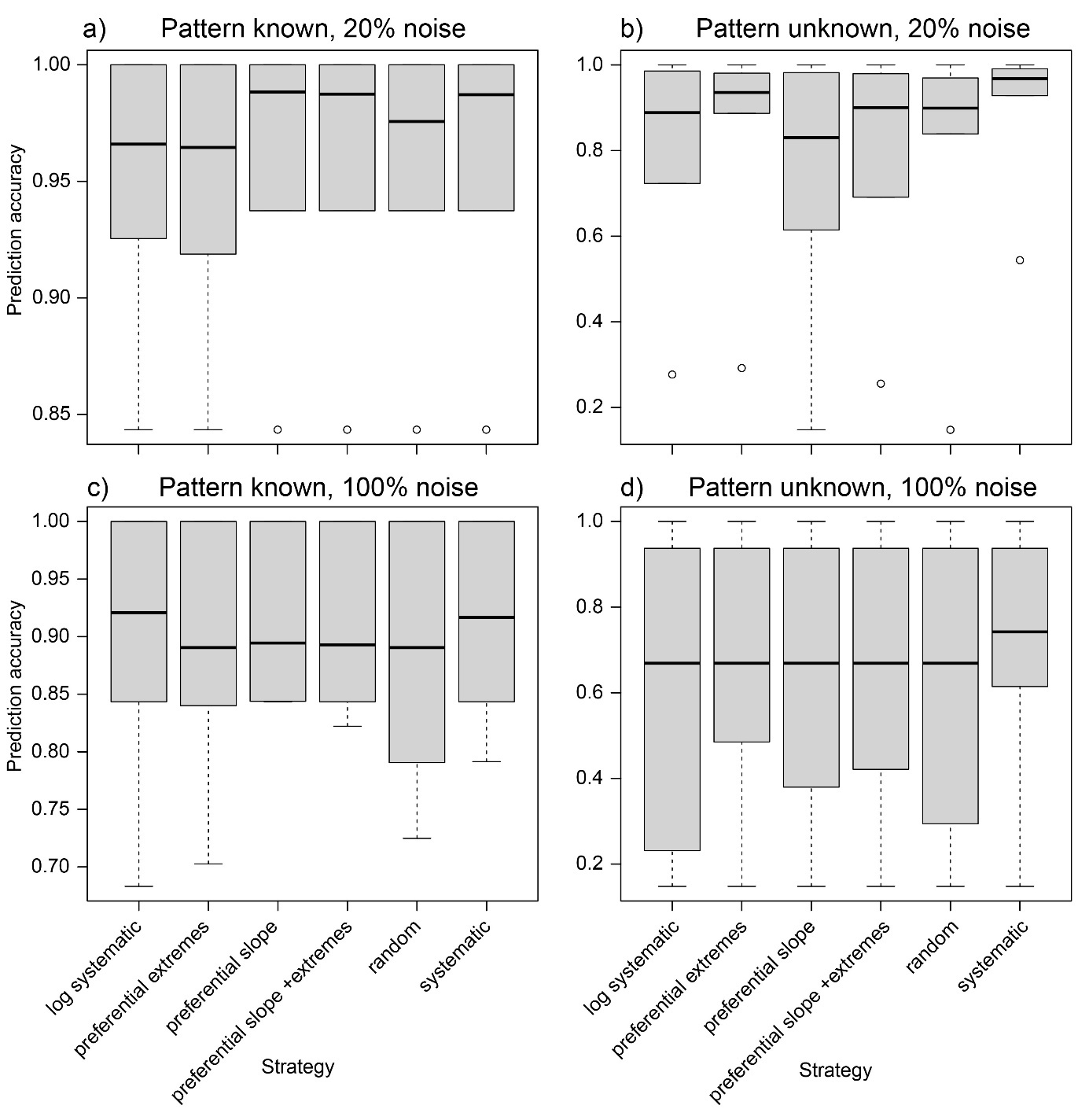


***Figure S5.*** *Differences in prediction accuracy between the different sampling strategies. Prediction accuracy is measured as multiple R^2^. Results are shown for scenarios, where the underlying response pattern is* a priori *known (a and c for 20% and 100% noise, respectively) as well as scenarios with unknown response patterns (b and d for 20% and 100% noise, respectively). Note that the y-axes cover different ranges.*


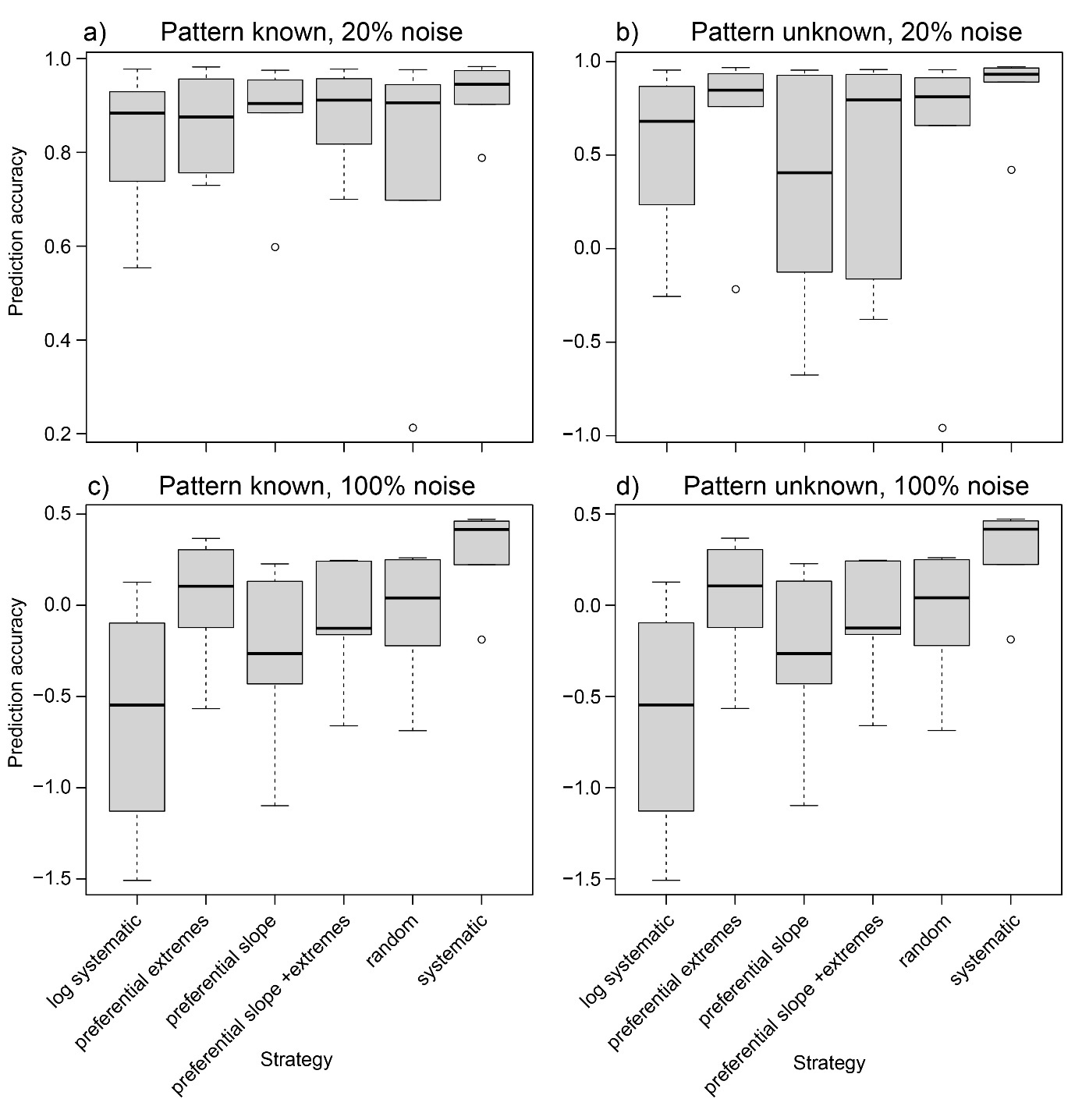


***Figure S6.*** *Differences in prediction accuracy between the different sampling strategies. Prediction accuracy is measured as Chalcraft’s prediction success. Results are shown for scenarios, where the underlying response pattern is a priori known (a and c for 20% and 100% noise, respectively) as well as scenarios with unknown response patterns (b and d for 20% and 100% noise, respectively). Note that the y-axes cover different ranges.*


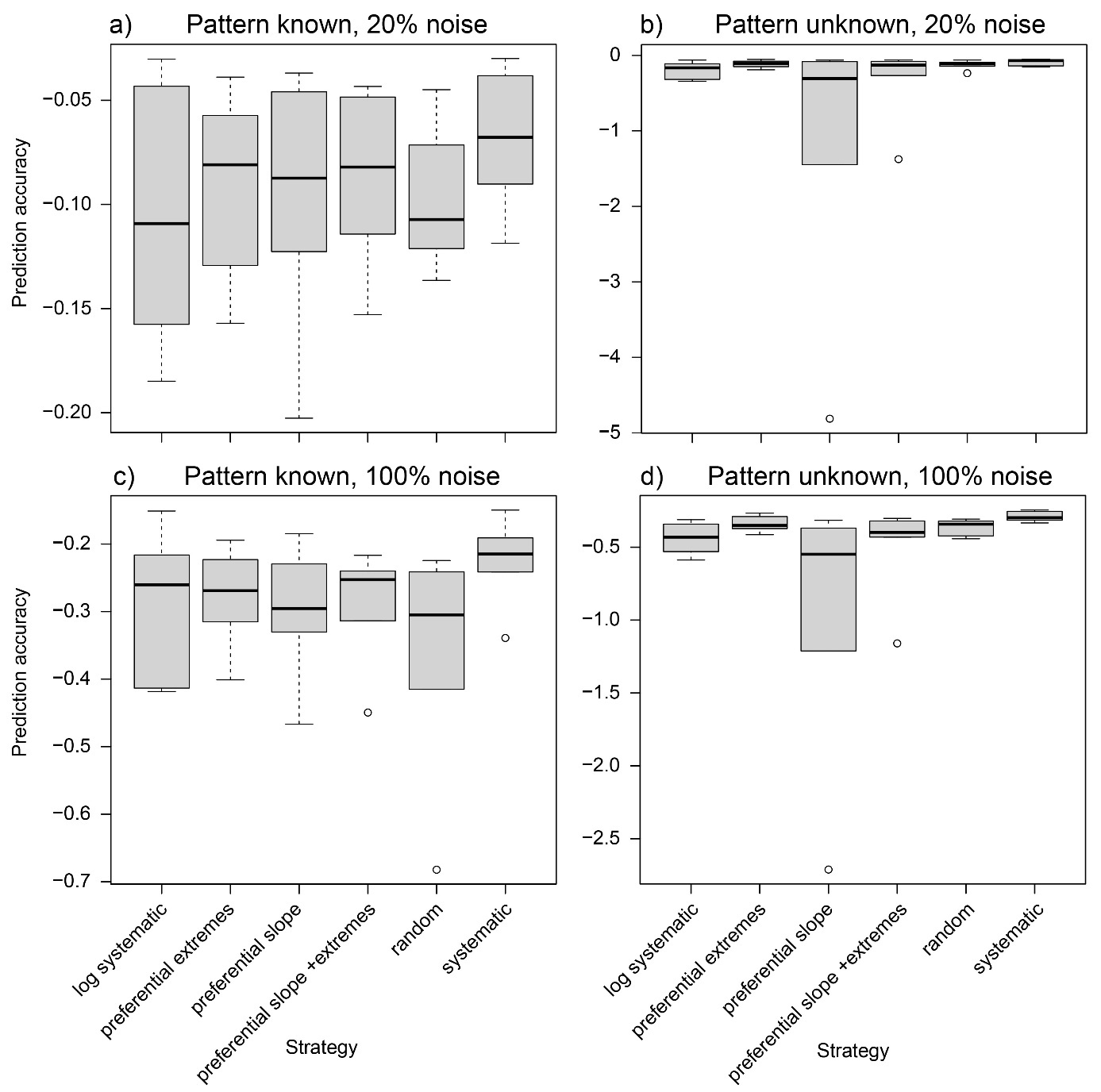


***Figure S7.*** *Differences in prediction accuracy between the different sampling strategies. Prediction accuracy is measured as RMSE (negative values). Results are shown for scenarios, where the underlying response pattern is* a priori *known (a and c for 20% and 100% noise, respectively) as well as scenarios with unknown response patterns (b and d for 20% and 100% noise, respectively). Note that the y-axes cover different ranges.*


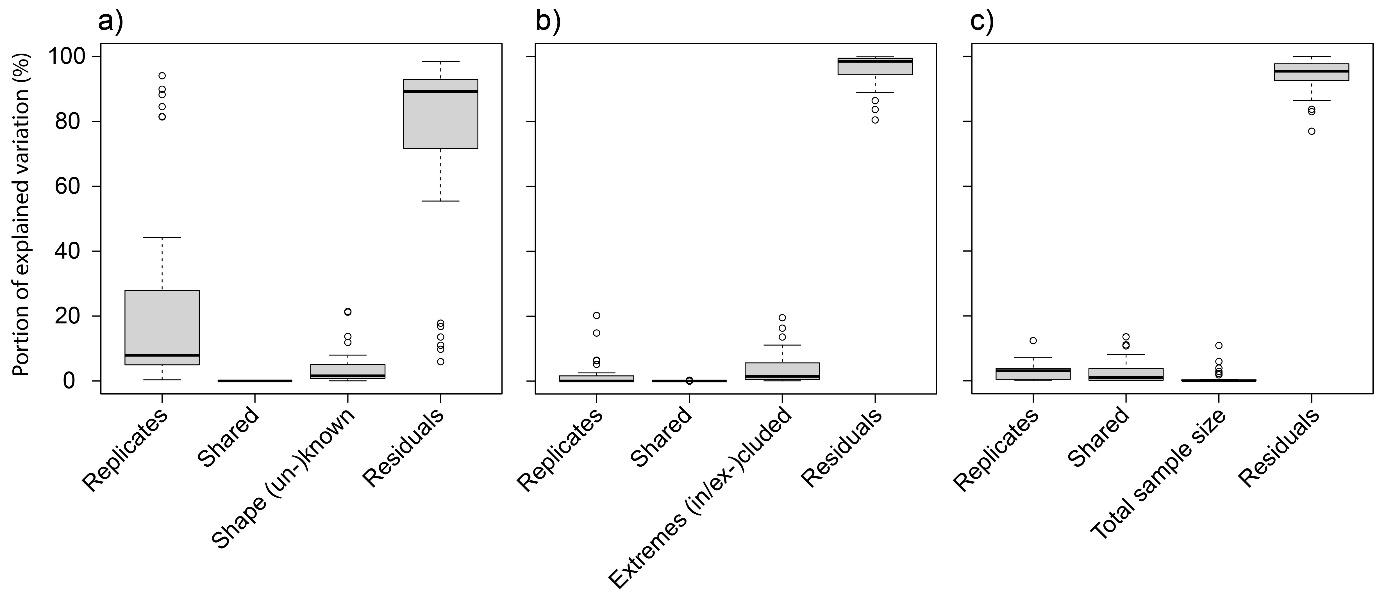


***Figure S8.*** *Relative contributions of replicates,* a priori *knowledge of the underlying response shape, length of the sampled gradient (i.e. extremes in-/excluded) and total sample size for explaining prediction accuracy (i.e. multiple R^2^). Depicted are the relative contributions (i.e. portion of explained variation) for each single explanatory and their interactions (i.e. shared). The outcome is shown for the effects of replicates**a priori *knowledge (a), replicates*gradient length (b) and replicates*total sample size (c). The tested, linear mixed effect models correspond to model 2 to 4 defined in the methods section. Residuals correspond to the portion of unexplained variation. Based on 20 % of random variation (noise) in the response (i.e. prediction accuracy).*


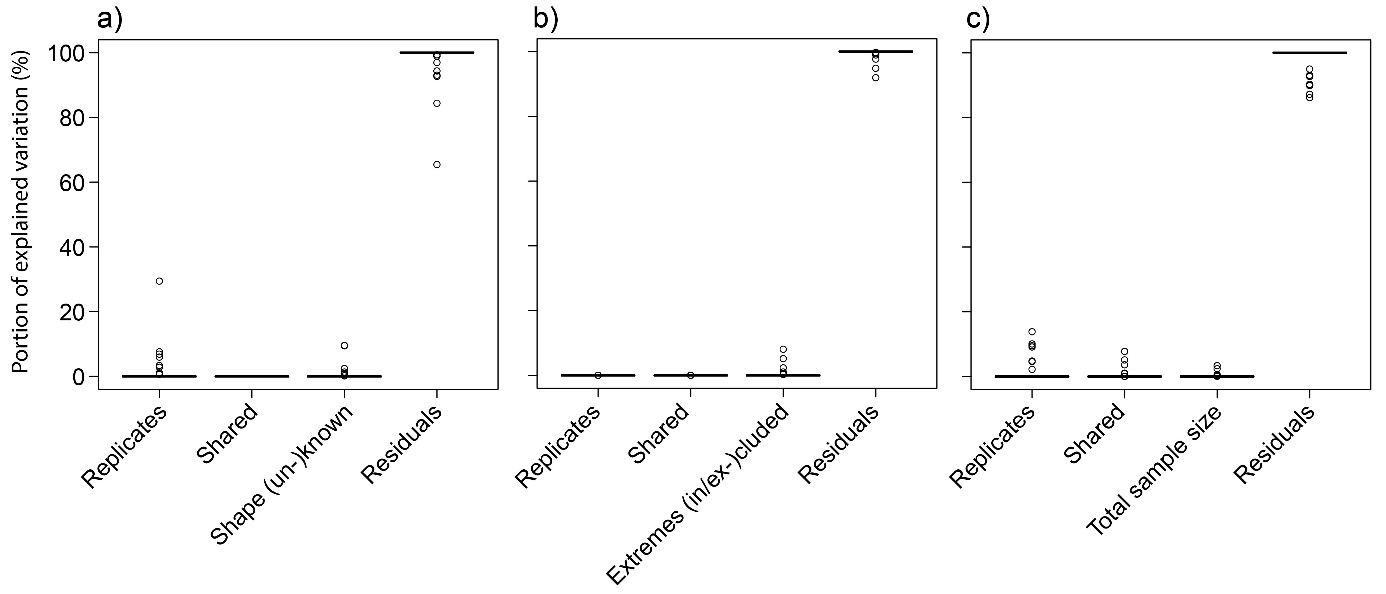


***Figure S9.*** *Relative contributions of replicates,* a priori *knowledge of the underlying response shape, length of the sampled gradient (i.e. extremes in-/excluded) and total sample size for explaining prediction accuracy (i.e. Chalcraft’s prediction accuracy). Depicted are the relative contributions (i.e. portion of explained variation) for the single explanatories and their interactions (i.e. shared). The outcome is shown for the effects of replicates**a priori *knowledge (a), replicates*gradient length (b) and replicates*total sample size (c). The tested, linear mixed effect models correspond to model 2 to 4 defined in the methods section. Residuals correspond to the portion of unexplained variation. Based on 20 % of random variation (noise) in the response (i.e. prediction accuracy).*


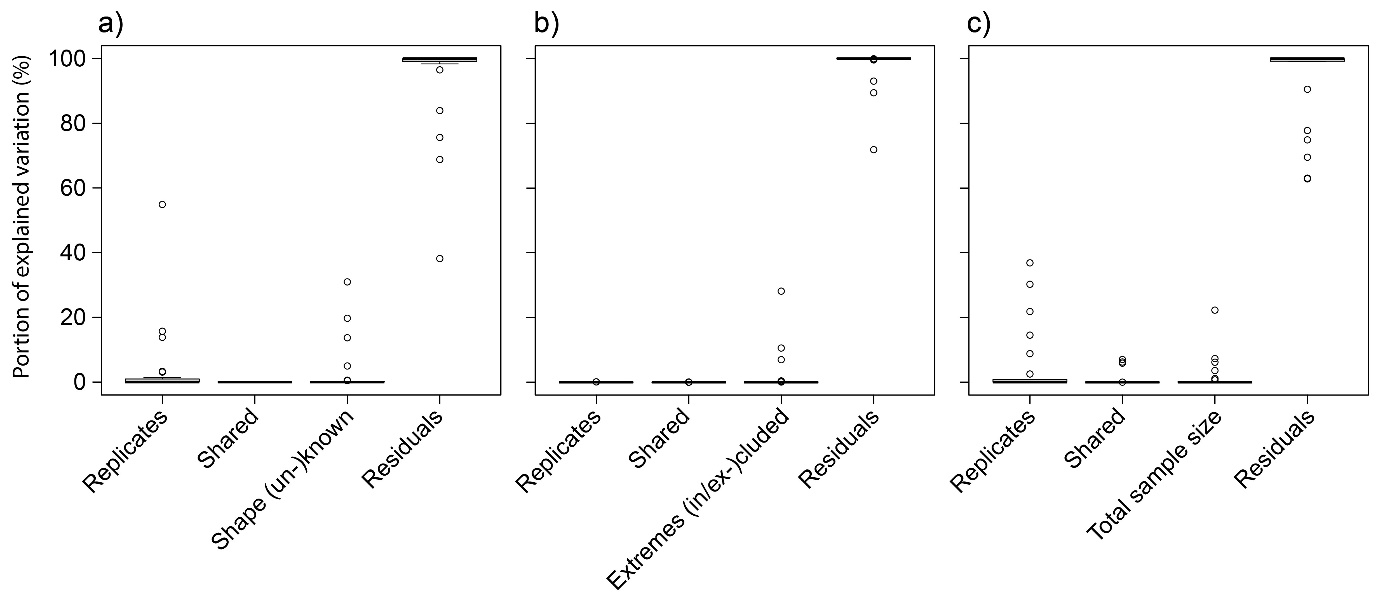


**Figure S10.** *Relative contributions of replicates,* a priori *knowledge of the underlying response shape, length of the sampled gradient (i.e. extremes in-/excluded) and total sample size for explaining prediction accuracy (i.e. negative RMSE). Depicted are the relative contributions (i.e. portion of explained variation) for the single explanatories and their interactions (i.e. shared). The outcome is shown for the effects of replicates**a priori *knowledge (a), replicates*gradient length (b) and replicates*total sample size (c.) The tested, linear mixed effect models correspond to model 2 to 4 defined in the methods section. Residuals correspond to the portion of unexplained variation. Based on 20 % of random variation (noise) in the response (i.e. prediction accuracy).*


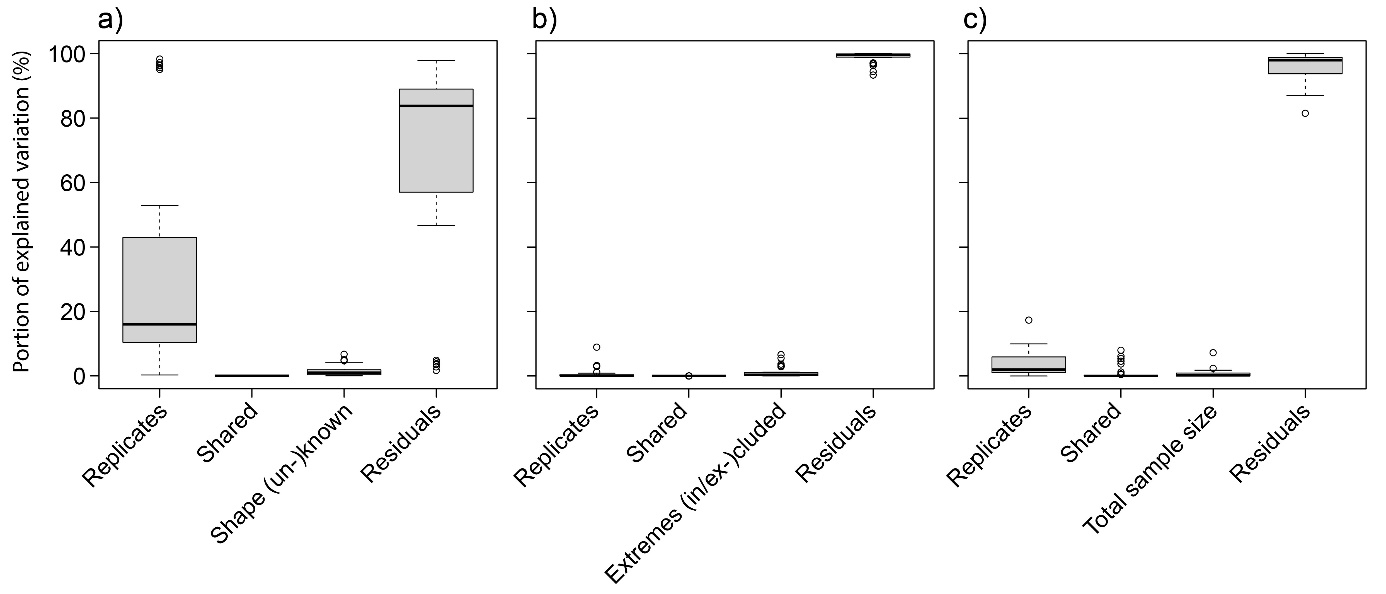


***Figure S11.*** *Relative contributions of replicates, a priori knowledge of the underlying response shape, length of the sampled gradient (i.e. extremes in-/excluded) and total sample size for explaining prediction accuracy (i.e. multiple R^2^). Depicted are the relative contributions (i.e. portion of explained variation) for each single explanatory and their interactions (i.e. shared). The outcome is shown for the effects of replicates**a priori *knowledge (a), replicates*gradient length (b) and replicates*total sample size (c). The tested, linear mixed effect models correspond to model 2 to 4 defined in the methods section. Residuals correspond to the portion of unexplained variation. Based on 100 % of random variation (noise) in the response (i.e. prediction accuracy).*


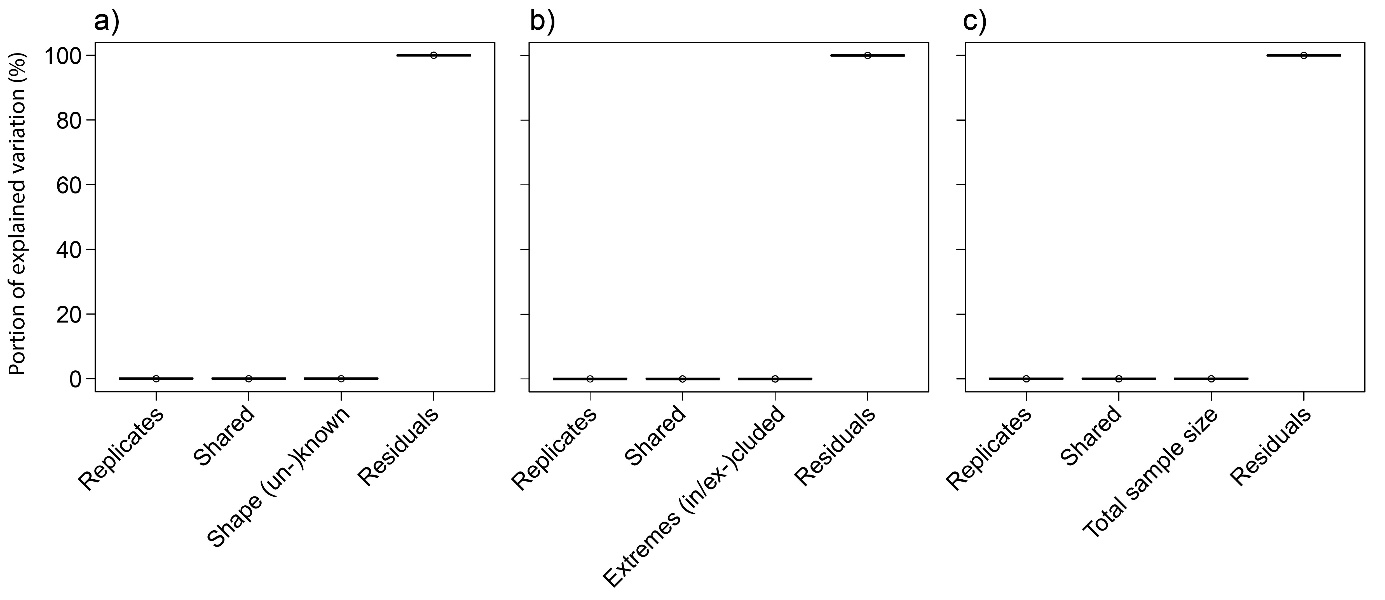


***Figure S12.*** *Relative contributions of replicates, a priori knowledge of the underlying response shape, length of the sampled gradient (i.e. extremes in-/excluded) and total sample size for explaining prediction accuracy (i.e. Chalcraft’s prediction accuracy). Depicted are the relative contributions (i.e. portion of explained variation) for each single explanatory and their interactions (i.e. shared). The outcome is shown for the effects of replicates**a priori *knowledge (a), replicates*gradient length (b) and replicates*total sample size (c). The tested, linear mixed effect models correspond to model 2 to 4 defined in the methods section. Residuals correspond to the portion of unexplained variation. Based on 100 % of random variation (noise) in the response (i.e. prediction accuracy).*


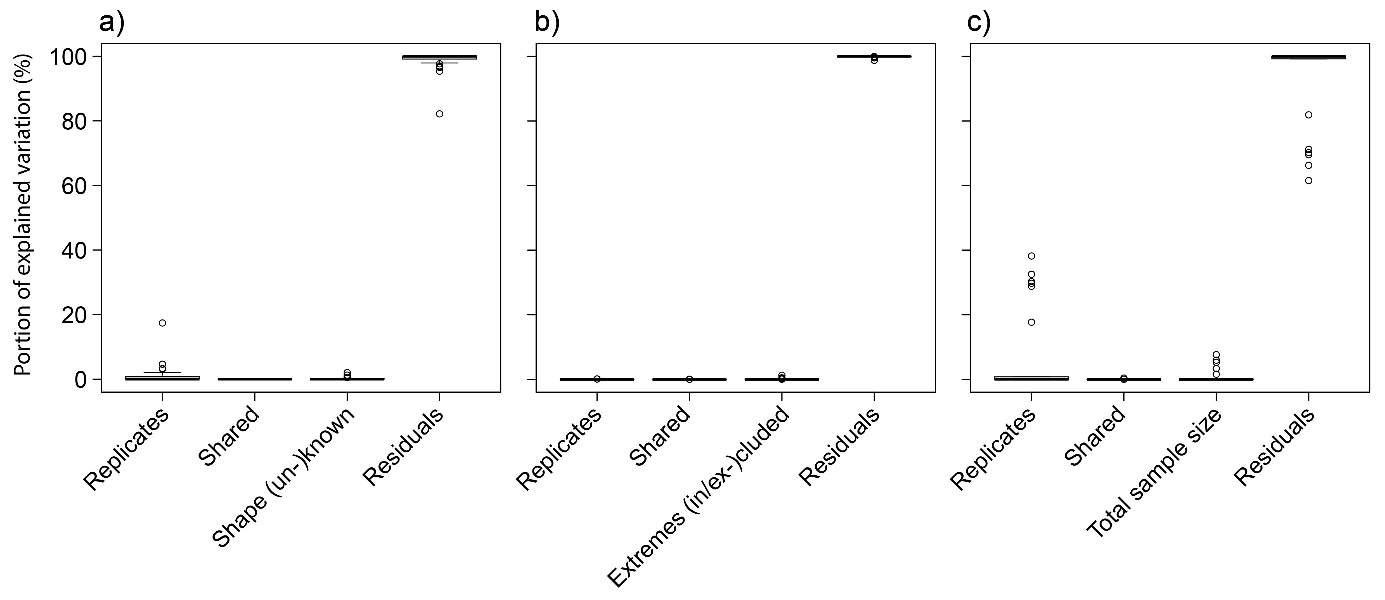


**Figure S13.** *Relative contributions of replicates, a priori knowledge of the underlying response shape, length of the sampled gradient (i.e. extremes in-/excluded) and total sample size for explaining prediction accuracy (i.e. negative RMSE). Depicted are the relative contributions (i.e. portion of explained variation) for the single explanatories and their interactions (i.e. shared). The outcome is shown for the effects of replicates**a priori *knowledge (a), replicates*gradient length (b) and replicates*total sample size (c). The tested, linear mixed effect models correspond to model 2 to 4 defined in the methods section. Residuals correspond to the portion of unexplained variation. Based on 100 % of random variation (noise) in the response (i.e. prediction accuracy).*


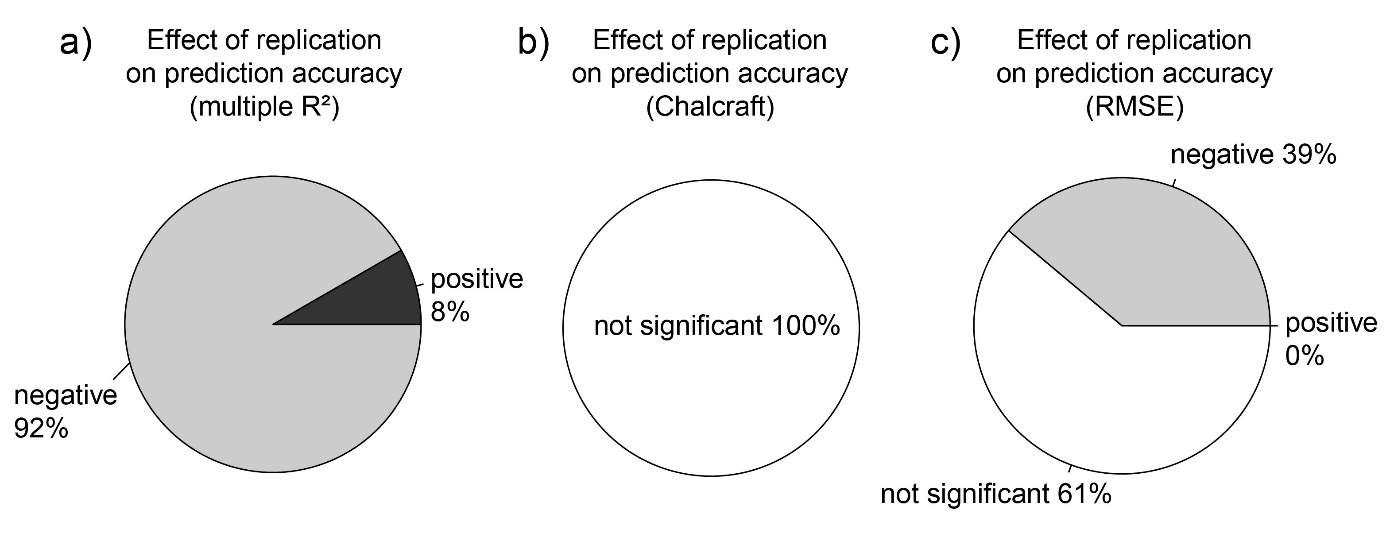


***Figure S14.*** *Effect of replication on prediction accuracy and a given number of samples. Effects were quantified for prediction accuracy measures based on multiple R^2^ (a), negative RMSE (b) and Chalcraft’s prediction success (c) for 100% noise.* A priori *knowledge of the underlying response shape was accounted for in a linear mixed effect model (Model 3 in Table 1).*


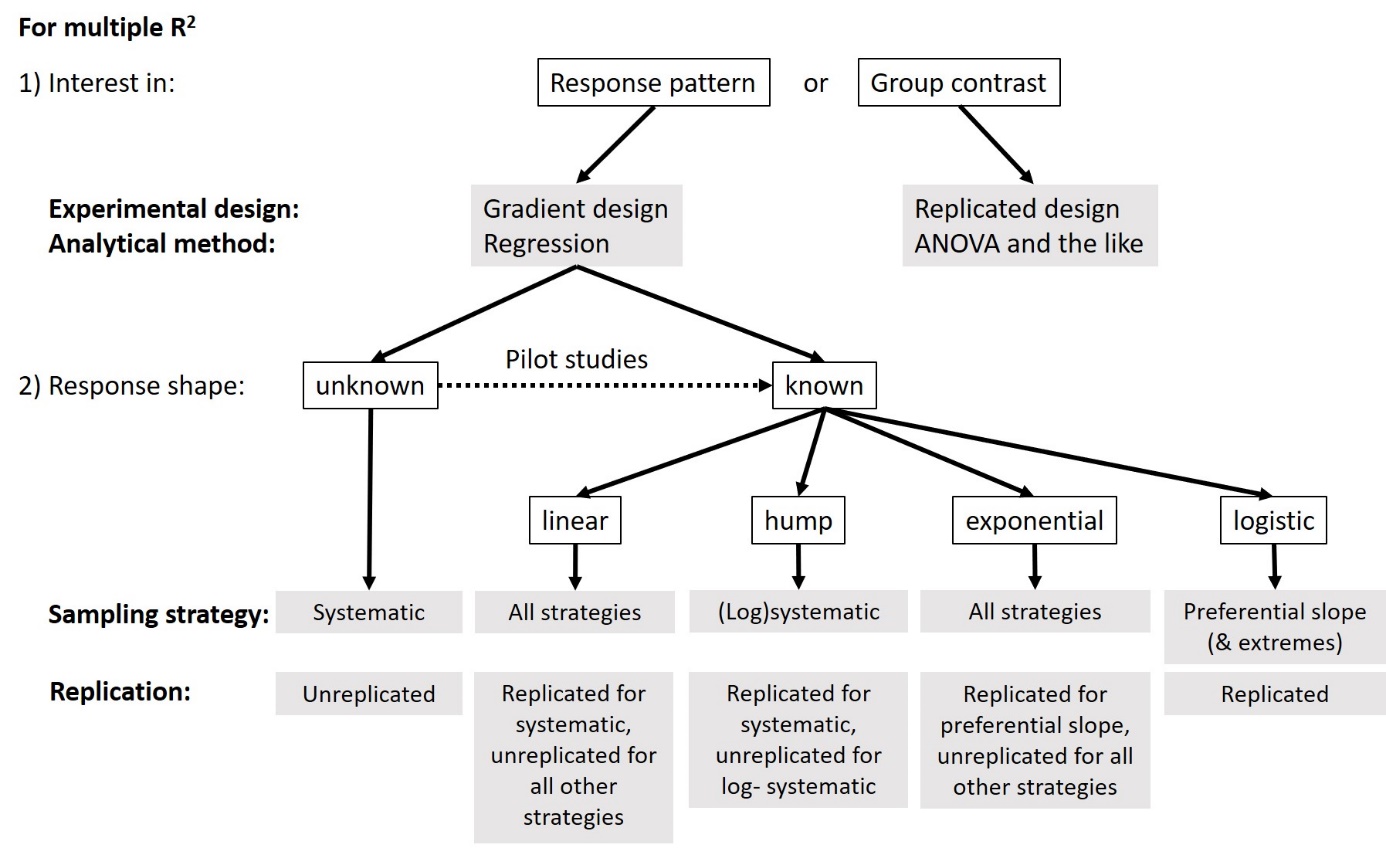


***Figure S15.*** *Decision tree for ecological experimenters based on general considerations and conclusions drawn from simulation results based on multiple R^2^.*


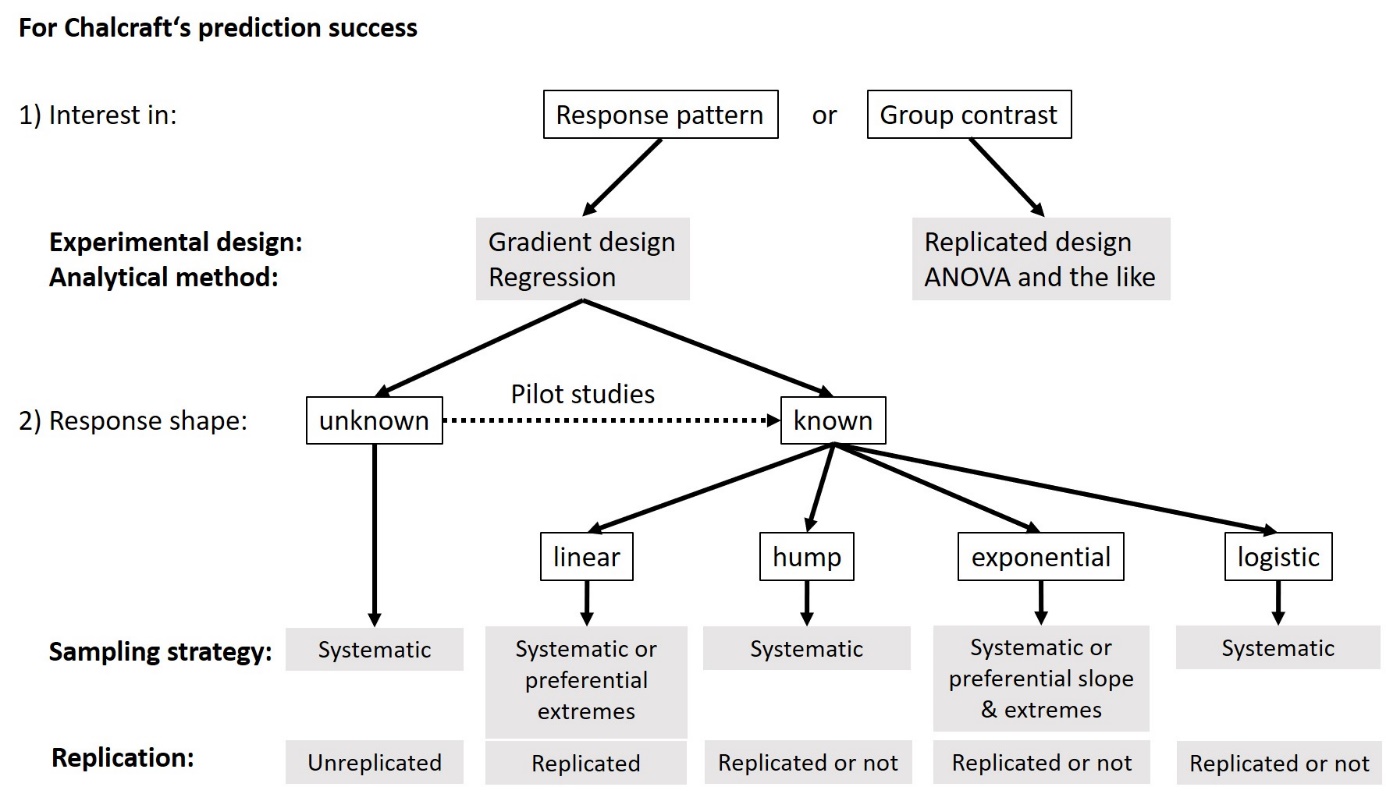


***Figure S16.*** *Decision tree for ecological experimenters based on general considerations and conclusions drawn from simulation results based on Chalcraft’s prediction success.*


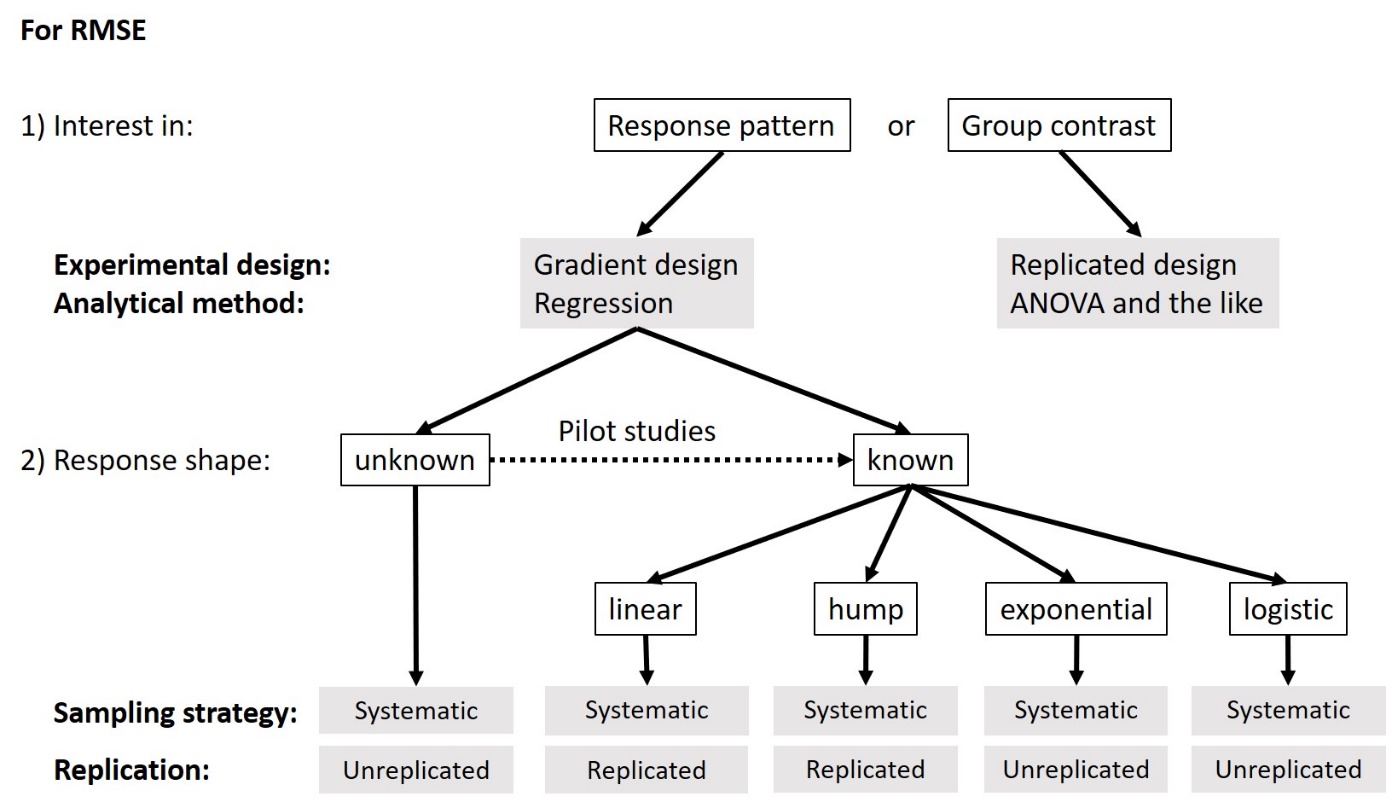


***Figure S17.*** *Decision tree for ecological experimenters based on general considerations and conclusions drawn from simulation results based on RMSE (negative values).*
